# Serotonin transporter gene polymorphism may be associated with functional dyspepsia in a Japanese population

**DOI:** 10.1186/1471-2350-12-88

**Published:** 2011-06-29

**Authors:** Fumihiko Toyoshima, Tadayuki Oshima, Shigemi Nakajima, Jun Sakurai, Junji Tanaka, Toshihiko Tomita, Kazutoshi Hori, Takayuki Matsumoto, Hiroto Miwa

**Affiliations:** 1 Divisions of Upper Gastroenterology, Department of Internal Medicine, Hyogo College of Medicine, 1-1 Mukogawa-cho, Nishinomiya, Hyogo 663-8501, Japan; 2 Departments of Medicine, Gastroenterology and Health Care, Social Insurance Shiga Hospital, 16-1 Fujimidai, Otsu, Shiga 520-0846, Japan; 3 Divisions of Lower Gastroenterology, Department of Internal Medicine, Hyogo College of Medicine, 1-1 Mukogawa-cho, Nishinomiya, Hyogo 663-8501, Japan

## Abstract

**Background:**

Although familial clustering of functional dyspepsia (FD) has been reported, the role of genetics in the susceptibility to FD is still not well understood. In the present study, the association between serotonin transporter (SERT) gene (*SLC6A4*) polymorphism and FD was explored.

**Methods:**

Subjects were divided into either a postprandial distress syndrome (PDS) group or an epigastric pain syndrome (EPS) group according to the Rome III criteria. The healthy controls were those who had visited a hospital for an annual health check-up. The presence of the *SLC6A4 *promoter polymorphism, *5-hydroxytryptamin transporter gene linked polymorphic region *(*5-HTTLPR*), was then evaluated, and logistic regression analysis was used to test all variables.

**Results:**

The *5-HTTLPR *genotype distribution was 448 SS, 174 SL, and 24 LL in controls and 30 SS, 20 SL, and 3 LL in FD subjects. No significant correlation was found between the *5-HTTLPR *genotype and FD. When the genotypes and subtypes of FD were exploratory evaluated, the SL genotype was significantly associated with PDS [odds ratio (OR) = 2.24, 95% confidence interval (CI); 1.16-4.32, *P *= 0.034 after Bonferroni correction] compared to the SS genotype adjusted for sex and age. Comparison of the SS genotype with the SL/LL genotype also showed a significant association of genotype with PDS (OR = 2.32, 95% CI; 1.23-4.37, *P *= 0.009).

**Conclusion:**

The present results suggest that *5-HTTLPR *L allele may influence the susceptibility to PDS.

## Background

Functional dyspepsia (FD) is characterized by the presence of symptoms thought to originate in the gastroduodenal tract in the absence of any organic or systemic disease that explains the symptoms [1]. The precise pathophysiology of the functional gastrointestinal disorders is still unknown. However, several pathophysiological mechanisms have been described as possible etiological factors: visceral hypersensitivity [2,3], impaired proximal gastric accommodation [4], delayed or early gastric emptying [5], dysfunction of the autonomic nervous system [6], and underlying psychiatric disturbances [7]. Although risk factors for FD, including age, sex, *Helicobacter pylori *infection, smoking, and psychological disturbances, have also been reported, the data are inconclusive [8,9].

There is increasing evidence that susceptibility to functional gastrointestinal disorders is influenced by hereditary factors. We have reported the association between *G-protein β3 subunit 825TT *genotype and EPS-like dyspepsia [10]. *IL-17F 7488T/C*, *migration inhibitory factor G173C *[11], *catechol-o-methyltransferase gene val158met *[12], *779 TC *of *CCK-1 intron 1 *[13], *cyclooxygenase-1 T1676C *[14], *p22 phagocyte oxidase C242T *[15], *transient receptor potential cation channel*, *subfamily V*, *member 1 G315C *[16], and *regulated upon activation normal T cell expressed and secreted C-28G *polymorphisms have also been reported to be associated with the development of FD or dyspeptic symptoms in the Japanese population [17].

Serotonin (5-hydroxytryptamin, 5-HT) is an important signaling molecule affecting gastrointestinal motor and sensory functions. Ninety-five percent of the body's 5-HT is synthesized in enterochromaffin cells in the gut. 5-HT binds to 7 subclasses of serotonergic receptors differentiated on the basis of structure, molecular mechanisms, and function [18]. To terminate serotonergic action, re-uptake occurs by the serotonin transporter protein (SERT) from the synaptic cleft. This protein is encoded by a single gene (*SLC6A4*) on chromosome 17q11 and is composed of 14 exons [19]. There is a 44-bp insertion/deletion in the 5'-flanking promoter region (*5-HT transporter gene linked polymorphic region*, *5-HTTLPR*), which creates a short and a long allele (S and L allele). The S allele of *5-HTTLPR *has been associated with lower transcriptional efficiency than the L allele [20].

Several studies have investigated the association between this functional polymorphism and various complex behavioral traits and disorders, including anxiety [20], major depression [21], suicide [22], smoking behavior [23], alcohol dependence [24], and irritable bowel syndrome [25-32]. Only two studies have explored the association between *5-HTTLPR *polymorphism and FD. However, there were no significant associations between them [33,34]. We thought it necessary to undertake a large-scale general population study. In this study, the relationship between the *5-HTTLPR *polymorphism and FD was investigated in Japanese patients, with subjects who underwent annual health check-up acting as controls.

## Methods

### Subjects

A total of 77 subjects with uninvestigated dyspepsia at Hyogo College of Medicine from June 2006 to June 2010 were enrolled. All subjects were Japanese and completed an original self-administered questionnaire that assessed symptoms of dyspepsia, gastroesophageal reflux disease (GERD), and irritable bowel syndrome (IBS) according to the ROME III criteria. Dyspeptic symptoms were defined as pain or discomfort in the upper abdomen for the last 3 months, with symptom onset at least 6 months prior to the check-up. Postprandial distress syndrome (PDS) was defined as postprandial fullness and early satiation, and epigastric pain syndrome (EPS) was defined as epigastric pain and epigastric burning for more than 6 months with symptoms. Age, sex, smoking, alcohol consumption, previous upper gastrointestinal studies, and previous medication were also recorded. Subjects who consumed alcohol more than 4 days a week, regardless of the amount, were considered to have a drinking habit. Smoking was defined as smoking any number of cigarettes daily. As judged by the physician, blood tests and further examinations such as esophagogastroduodenoscopy (EGD) and abdominal ultrasonography (US) were performed. A total of 24 subjects who had significant EGD or US findings, such as peptic ulcer disease, malignancies, gall stone, or abnormal results in laboratory parameters including ALT or WBC, was excluded from this study. Finally, 53 subjects were diagnosed as having FD, of which only 3 subjects also overlapped with IBS according to the ROME III criteria. As controls, people visiting the Healthcare Center of Social Insurance Shiga Hospital for an annual health check-up were asked to participate in the study. About 80% of those who underwent an annual health check-up from December 2007 to April 2008 agreed to participate in the study. Of the 1000 subjects who completed the same questionnaire, 646 subjects who did not have any symptoms of dyspepsia were included in this study. This study was approved by the ethics committees of both the Hyogo College of Medicine and the Social Insurance Shiga Hospital, and written informed consent was obtained from all participants.

### Genotyping

DNA was isolated from whole blood from 699 participants by the alkaline lysis method using the QIAamp DNA Blood Maxi Kit (Qiagen Inc., Valencia, CA). The polymorphisms in the *5-HTTLPR *were identified by polymerase chain reaction (PCR)-based fragment length polymorphisms. Polymorphisms were confirmed by direct sequencing. Oligonucleotide primers flanking the *5-HTTLPR *long polymorphic region corresponding to the nucleotide positions 1651 to 1670 (sense 5' GCCGCTCTGAATGCCAGCAC 3') and 2242 to 2265 (antisense 5' GGAGGAACTGACC-CCTGAAAACTG 3') were used to generate 528- and/or 572-base-pair PCR-amplified fragments. Both sequences were obtained from the GenBank/EBI Data Bank, accession number X76753. PCR amplification was performed in a final volume of 20 μl, consisting of 0.2 μg of genomic DNA, 400 μmol/L deoxyribonucleotides, and 0.2 μmol/L of each primer. Because of the high guanine and cytosine (GC) content in the amplified region of the SERT gene *(SLC6A4)*, the PCR reactions were performed using the TaKaRa La Taq polymerase (0.8 U/reaction) with GC Buffer I (TaKaRa Biomedicals, Shiga, Japan). After denaturing all DNA samples at 94°C for 1 min, cycling conditions were set at 30 cycles of 94°C for 30 s, 60°C for 30 s, and 72°C for 2 min, with a 10-min final cycle extension at 72°C. To determine the presence of length variations of the alleles, amplified products were electrophoresed on 2.0% agarose and visualized by ethidium bromide staining.

### Statistical Analysis

The median and range in each group were calculated, and differences were compared using the nonparametric Mann-Whitney U-test. Differences between *5-HTTLPR *genotypes and sex, smoking, or drinking were compared by Fisher's exact test. The distribution of alleles at each locus was assessed using the χ^2 ^statistic of the Hardy-Weinberg equilibrium. *5-HTTLPR *genotypes were compared between FD patients and controls, and the association between specific types of FD and *5-HTTLPR *polymorphism was assessed. A logistic regression analysis was performed to test the influence of several factors in the association between the *5-HTTLPR *polymorphism genotype distribution and FD. *P *< 0.05 was considered significant. The Bonferroni correction was applied for multiple testing of each genotype.

### Power of the Study

In this study, the potential association of symptoms of dyspepsia with *5-HTTLPR *genotypes was assessed. In the healthy Japanese population, approximately 30% are expected to have the SL/LL genotype. Assuming that approximately 5% of subjects have symptoms of dyspepsia, a 20% increase in the prevalence of a genotype would be of clinical relevance. Thus, setting α = 0.05 and β = 0.80, 977 asymptomatic controls and 52 subjects with FD would be sufficient to identify a clinically relevant difference. The actual number of enrolled subjects (53 FD cases and 646 controls) has a power of 80% to detect the assumed difference. For the subgroup analyses for EPS (39 cases), and PDS (42 cases), the power to identify the assumed difference is 67%, and 70%, respectively.

## Results

Participant demographics and *5-HTTLPR *genotype distribution are summarized in Table 1. The median age of the subjects with dyspepsia and the non-dyspeptic controls was 52 (range, 21-82) and 45 (range, 19-78) years (*P *= 0.002). No significant bias was found between the groups for sex, smoking habit, and drinking habit. The *5-HTTLPR *genotype distribution in all subjects in this study was 478 SS (68.4%), 194 SL (27.8%), and 27 LL (3.9%); this distribution was compatible with the Hardy-Weinberg equilibrium (*P *= 0.195). The *5-HTTLPR *genotype distribution was 448 SS (69.3%), 174 SL (26.9%), and 24 LL (3.7%) in subjects without dyspepsia (controls), and 30 SS (56.6%), 20 SL (37.7%), and 3 LL (5.7%) in subjects with FD; both distributions were also compatible with the Hardy-Weinberg equilibrium. The distribution of the SS/SL/LL genotypes in the healthy controls was similar to other reports for Japanese populations [35,36]. The distribution of allele and genotype frequencies did not differ significantly between males and females.

**Table 1:** Participant demographics and genotype distributions

	Controls (n = 646)	FD, total (n = 53)	*P*^2^	EPS (n = 39)^4^	*P*^2^	PDS (n = 42)^4^	*P*^2^
Age (years)^1^	45 (19-78)	52 (21-82)	0.002	50 (21-82)	0.021	51 (21-82)	0.015
Sex (male%)	50.5	49.1	0.887	41.0	0.323	52.4	0.874
Smoking (%)	32.7	28.3	0.647	23.1	0.289	31.0	1.000
Drinking (%)	29.6	32.1	0.755	30.8	0.858	33.3	0.604
*5-HTTLPR *genotype (%)							
SS	69.3	56.6		61.5		50.0	
SL	26.9	37.7		30.8		42.9	
LL	3.7	5.7	0.117	7.7	0.257	7.1	0.019
*P *for HWE^3^	0.173	0.889		0.405		0.746	

^1^Data are shown as median (range).

^2^*P *vs. controls by Mann-Whitney U-test (for age) or Fisher's exact test (for other variables).

^3^*P *for deviation from Hardy-Weinberg equilibrium (HWE).

^4^Twenty eight subjects are simultaneously classified EPS and PDS phenotype.

FD, functional dyspepsia; EPS, epigastric pain syndrome; PDS, postprandial distress syndrome

The association of genotypes with the overall FD phenotype compared with asymptomatic controls was not significant (*P *= 0.117). Thirty-nine subjects had EPS, whereas 42 had PDS; 28 subjects simultaneously had EPS and PDS (Table 1). When the subtypes of FD and the genotypes were exploratory evaluated, a significant association of genotype in PDS subjects compared to that in control was detected (*P *= 0.019). On the other hand, no association of genotype in subjects with the EPS phenotype compared to that in controls was detected. The odds ratios of the *5-HTTLPR *SL and LL genotypes relative to the SS genotype for the phenotypes of FD, EPS, PDS are shown in Table 2. Significant associations were detected for the SL genotypes in the PDS subtype [odds ratio (OR) = 2.24, 95% confidence interval (CI); 1.16-4.32, *P *= 0.033 after Bonferroni correction] adjusted for sex and age (Table 2). Comparison of the SS genotype with the SL/LL genotype also showed a significant association of genotype (OR = 2.32, 95% CI; 1.23-4.37, *P *= 0.009) (Table 2). However, there was no significant association of genotype detected in the FD and EPS phenotypes.

**Table 2:** Risk of dyspepsia according to *5-HTTLPR *genotypes

	FD, total (n = 53)			EPS (n = 39)^1^			PDS (n = 42)^1^		
						
	OR	(95%CI)	*P* uncorrected (corrected)	OR	(95%CI)	*P* uncorrected (corrected)	OR	(95%CI)	*P* uncorrected (corrected)
*5-HTTLPR *genotype									
SS	1	reference		1	reference		1	reference	
SL	1.75	(0.96-3.19)	0.672 (1.000)	1.33	(0.65-2.73)	0.443 (0.886)	*2.24	(1.16-4.32)	0.017 (0.033)
LL	2.12	(0.59-7.59)	0.250 (0.499)	2.50	(0.69-9.10)	0.164 (0.328)	3.02	(0.83-11.06)	0.094 (0.188)
*Recessive model*									
SS vs. SL+LL	1.79	(1.01-3.18)	0.472	1.46	(0.75-2.87)	0.268	*2.32	(1.23-4.37)	0.009

^1^Twenty eight subjects are simultaneously classified EPS and PDS phenotype.

FD, functional dyspepsia; EPS, epigastric pain syndrome; PDS, postprandial distress syndrome;

OR, sex- and age-adjusted odds ratio, vs. 646 controls, by a multiple logistic regression model; CI, confidence interval.

* Significant *P *< 0.05

## Discussion

In the present study, the prevalence of the *5-HTTLPR *polymorphism was examined in dyspeptic patients in a Japanese population. Although the power was not enough for the analysis and the data are preliminary, the *5-HTTLPR *L allele may be associated with an increased risk of PDS. Camilleri et al. and van Lelyveld et al. reported that the *5-HTTLPR *polymorphism was not associated with dyspepsia in a population in the US and the Netherlands [33,34]. The SERT mRNA expression in the gastric specimens of pediatric age FD patients was not significantly different compared with healthy controls [37]. These contrasting observations may be explained by differences in the genotypic composition of populations in different countries with different racial groups. In fact, the frequency of the LL subtype is lower in the Japanese population than in Caucasian populations [35,36]. In addition, the definition of FD or sample selection may also affect the outcome. Moreover, the effect of type II error cannot be excluded in relatively small sample sizes. Another limitation of this study was that the ages were different in the groups. However, age adjustment was performed in genotype analysis using logistic regression.

The SERT is the target of selective serotonin re-uptake inhibitors (SSRI), which are widely used as antidepressants. Although SSRIs have been reported to have a benefit in IBS [38], few trials have focused on FD treatment. Any evidence of effectiveness is weak to nonexistent. Wu et al. conducted an open clinical trial with 40 FD patients. They reported that fluoxetine improved symptom scores in depressive FD patients [39]. Van Kerkhoven et al. conducted a randomized controlled trial with venlafaxine in 160 FD patients and showed no significant difference compared with placebo [40].

The results of the present study suggest the hypothesis that the *5-HTTLPR *L allele may be associated with PDS. Based on *in vitro *studies of *5-HTTLPR *polymorphism function, excessive transcription of SERT in *5-HTTLPR *L allele would be expected to lead to higher 5-HT reuptake activity and decreased levels of 5-HT in the synaptic cleft [20], which in turn might result in decreased gut motility and secretion, subsequently causing postprandial fullness and early satiety, which are PDS symptoms. Actually after excluding subjects with both EPS and PDS, the association of SL genotype for the development of PDS was stronger (data not shown). However, the power was too small to conclude this from the present study. Furthermore, multiple testing may also affect the results. Therefore, we cannot exclude the effect of type I error.

The secreted 5-HT affects at least 30 subtypes of 7 main subclasses of serotonergic receptors that cause various effects on the gut or brain. There are several reports about the association between specific serotonergic receptors and dyspepsia. We have reported that the 5HT1A agonist tandospirone improved abdominal symptom scores, including upper abdominal pain and discomfort [41]. On the other hand, Tack et al. reported that a 5HT1A agonist failed to improve symptoms, visceral hypersensitivity, or gastric accommodation compared with placebo [42]. Boeckxstaens et al. also reported that a 5HT1A agonist had no effect on gastric fundus distension-evoked dyspeptic symptoms in healthy volunteers [43]. Talley et al. reported that alosetron, a 5HT3 antagonist, had potential benefit in relieving functional dyspepsia symptoms compared to placebo [44]. Maxton et al. and Kuo et al. also reported the effectiveness of alosetron for FD symptoms [45,46]. In addition, Vakil et al. reported in their meta-analysis that tegaserod, a 5HT4 agonist, showed significant benefit in dysmotility, as occurs in FD [47].

Some reports seem to support our hypothesis, but there are several conflicting results. Therefore, the role of SERT functional differences in the gut is still unclear. The physiological effects on gut function of *5-HTTLPR *polymorphism have been investigated in a report concerning IBS. However, there are many conflicting results [48].

Some studies have reported a sex relation in *5-HTTLPR *polymorphism. Yeo et al. reported a significant association between D-IBS and *5-HTTLPR *SS genotype in females [26]. A similar result for anxiety traits was reported by Mizuno et al. Females with the L/S genotype showed higher anxiety scores than males [49]. However, the sample size of this study was too small to conclude that there was a sex difference.

## Conclusion

We have exploratory shown that *5-HTTLPR *L allele may influence the susceptibility to PDS in a Japanese population. This is the first report that shows the possibility of a significant association between PDS and *5-HTTLPR *polymorphism. FD is a complex and heterogeneous disorder, and it is known that FD is not caused by a single gene. Additional studies with a further larger sample and different polymorphisms are mandatory to identify the genetic background that influences susceptibility to FD.

## List of abbreviations

FD: functional dyspepsia; 5-HT: 5-hydroxytryptamin (serotonin); SERT - serotonin transporter; *5-HTTLPR *- 5-HT transporter gene linked polymorphic region; GERD: gastroesophageal reflux disease; IBS- irritable bowel syndrome; PDS: postprandial distress syndrome; EPS: epigastric pain syndrome; EGD - esophagogastroduodenoscopy; US - ultrasonography; OR: odds ratio; CI: confidence interval.

## Competing interests

The authors declare that they have no competing interests.

## Authors' contributions

FT analyzed the data and wrote the paper. TO participated in the design of the study, and wrote part of the paper. SN, together with JS, JT, TT and KH, obtained the samples and the data. TM supervised the research project and the drafting of the manuscript. HM was responsible for the conception of the study and designed the study. All authors approved of the final manuscript prior to submission.

## Pre-publication history

The pre-publication history for this paper can be accessed here:

http://www.biomedcentral.com/1471-2350/12/88/prepub
